# Short wind pulses consistently change the morphology of roots, but not of shoots, across young plants of different growth forms

**DOI:** 10.1007/s44154-023-00123-z

**Published:** 2023-10-09

**Authors:** Johannes Heinze, Luise Werger, Michael Ogden, Thilo Heinken, Rainer Hoefgen, Ewald Weber

**Affiliations:** 1 Institute of Biochemistry and Biology, Biodiversity Research and Systematic Botany, University of Potsdam, Maulbeerallee 1, Potsdam, 14469 Germany; 2 Heinz Sielmann Foundation, Dyrotzer Ring 4, Wustermark (OT Elstal), 14641 Germany; 3 Department of Molecular Physiology, Max Planck Institute of Molecular Plant Physiology, Am Mühlenberg 1, Potsdam-Golm, 14476 Germany; 4 School of Biosciences, University of Melbourne, Melbourne, VIC 3010 Australia; 5 Institute of Biochemistry and Biology, General Botany, University of Potsdam, Maulbeerallee 3, Potsdam, 14469 Germany

**Keywords:** Wind stress, Leaf traits, Root traits, Growth form, Morphology

## Abstract

Wind is an environmental stimulus that stresses plants of all growth forms at all life-stages by influencing the development, architecture, and morphology of roots and shoots. However, comparative studies are scarce and no study directly investigated whether shoot and root morphological traits of trees, grasses and forbs differ in their response to short wind pulses of different wind intensity. In this study, we found that across species, wind stress by short wind pulses of increasing intensity consistently changed root morphology, but did not affect shoot morphological traits, except plant height in four species. Wind effects in roots were generally weak in tree species but consistent across growth forms. Furthermore, plant height of species was correlated with changes in specific root length and average diameter.

Our results indicate that short-pulse wind treatments affect root morphology more than shoot morphology across growth forms. They further suggest that wind stress possibly promotes root anchorage in young plants and that these effects might depend on plant height.

Wind is a ubiquitous environmental factor that, beside influencing plant growth and physiology, is known to impact the morphology (i.e., characteristics of individual plant organs) of plants (Gardiner et al. 2016). Although wind acts and stresses plants aboveground, plant responses appear both directly aboveground and indirectly belowground. For instance, wind effects were found to change morphological traits of shoots such as plant height (Feng et al. 2019) and leaf morphology (Anten et al. 2010) aboveground, but also root morphological traits (Werger et al. 2020) belowground. As abiotic stress, wind and its intensity impacts species of all growth forms, including trees, grasses and forbs, at all life-stages. However, these growth forms differ in their morphology both aboveground, for example in leaf morphology (Liu et al. 2019), and belowground in roots (Larson and Funk 2016). While much is known about natural and continuous effects of wind on plant morphology (de Langre 2008; Gardiner et al. 2016), much less is known about the effects of short wind stress.

To investigate whether shoot and root morphological traits of young plants of different growth forms differ in their response to wind, we performed a greenhouse experiment and treated plants with recurring wind pulses of different intensity (i.e., wind speed).

We found that growth forms differed in plant height and leaf dry matter content (LDMC), but however, that the factor ‘wind’ (i.e., short wind pulses of different intensity) did not affect leaf traits (i.e., leaf area (LA), specific leaf area (SLA) and LDMC; Table 1a). Within herbaceous plants, leaf traits of annuals and perennials were also not affected by wind stress (Table 1b). Overall, species showed a mostly similar lack of responses in leaf traits to wind (species x wind interaction: LA, SLA and LDMC > 0.1; Table 1c). Increasing wind pulse intensity decreased plant height in only four of 25 plant species in this experiment (Table 2a). While in trees and grasses the height of only one species each was affected by wind stress (*A. pseudoplatanus* in trees and *Z. mays* in grasses, Table 2a), in forbs two species (*T. dubium* and *B. napus*; Table 2a) drove a wind-induced decrease in plant height (*F*_2,123_ = 4.31, *P* = 0.015; Fig. 1d). This result, that the morphology of shoots, which are usually directly exposed to wind action (Gardiner et al. 2016), were mostly not affected by short wind pulses, is in contrast to common findings observed under continuous and longer wind action. For instance, previous studies reported a decrease of plant height with increasing wind intensity (e.g., Zhang et al. 2021). In our experiment, wind only affected plant height in four species, suggesting that the stress by wind pulses might have been too short and too weak to cause significant change. This might also be the reason why we did not observe changes in morphological leaf traits in our experiment, like other studies on wind effects on LA, LDMC and SLA (Retuerto and Woodward 1992; Anten et al. 2010).

**Table 1:** Results of ANOVAs that tested effects of (a) ‘Growth form’ (‘tree’, ‘grass’ and ‘forb’), (b) ‘Life history’ (‘annuals’ and ‘perennials’ within herbaceous plants) and (c) ‘Species’ (i.e., all 25 tested species; see Table 2a) – and ‘Wind pulse level’ (‘no wind’ vs. ‘medium’ vs. ‘high’ wind intensity) as well as their interactions on aboveground morphological traits [plant height, leaf area (LA), specific leaf area (SLA) and leaf dry matter content (LDMC)], root morphological traits [specific root length (SRL), specific root surface area (SRSA), average diameter (AD) and root tissue density (RTD)] and biomass production and allocation [shoot, root and total biomass as well as root:shoot ratio (R:S)]. The ‘numDf’ report the numerator degrees of freedom [i.e., the number of degrees of freedom for the factor tested] and ‘denDf’ the denominator degrees of freedom (i.e., the number of degrees of freedom accounting for the random effect; see Materials and methods section). Significant effects (*P* < 0.05) are reported in bold and marginal effects in italics (*P* < 0.1)

a) Model ‘Growth form’										
Aboveground traits	numDf	denDf	Height		LA		SLA		LDMC	
			*F*	*P*	*F*	*P*	*F*	*P*	*F*	*P*
Growth form (G)	2	22	8.76	**0.002**	0.12	0.885	2.53	0.104	13.21	**< 0.001**
Wind (W)	2	338	19.78	**< 0.001**	1.49	0.226	2.33	0.107	0.27	0.767
G x W	4	338	2.55	**0.039**	1.40	0.235	0.29	0.882	0.12	0.977
Root traits	numDf	denDf	SRL		SRSA		AD		RTD	
			*F*	*P*	*F*	*P*	*F*	*P*	*F*	*P*
Growth form (G)	2	22	4.33	**0.014**	1.35	0.262	12.84	**< 0.001**	16.25	**< 0.001**
Wind (W)	2	191	40.91	**< 0.001**	6.00	**0.003**	5.66	**0.004**	18.43	**< 0.001**
G x W	4	191	1.03	0.394	0.32	0.867	4.19	**0.003**	0.53	0.714
Biomass / allocation	numDf	denDf	Shoot		Root		Total		R:S	
			*F*	*P*	*F*	*P*	*F*	*P*	*F*	*P*
Growth form (G)	2	22	0.54	0.591	1.60	0.226	1.14	0.339	2.94	*0.075*
Wind (W)	2	338	0.98	0.377	0.60	0.547	0.08	0.921	1.02	0.362
G x W	4	338	0.56	0.693	1.76	0.153	1.45	0.217	2.00	*0.095*
b) Model ‘Life history’										
Aboveground traits	numDf	denDf	Height		LA		SLA		LDMC	
			*F*	*P*	*F*	*P*	*F*	*P*	*F*	*P*
Life history (L)	1	18	1.8	0.203	1.67	0.213	0.21	0.652	5.32	**0.034**
Wind (W)	2	273	4.27	**0.015**	1.28	0.278	1.63	0.101	0.41	0.662
L x W	2	273	1.79	0.143	0.95	0.387	0.06	0.938	0.18	0.839
Root traits	numDf	denDf	SRL		SRSA		AD		RTD	
			*F*	*P*	*F*	*P*	*F*	*P*	*F*	*P*
Life history (L)	1	18	1.12	0.211	2.47	0.134	0.51	0.486	0.06	0.799
Wind (W)	2	154	37.02	**< 0.001**	4.93	**0.008**	17.53	**< 0.001**	18.35	**< 0.001**
L x W	2	154	1.98	0.113	0.94	0.391	0.62	0.538	2,21	0.113
Biomass / allocation	numDf	denDf	Shoot		Root		Total		R:S	
			*F*	*P*	*F*	*P*	*F*	*P*	*F*	*P*
Life history (L)	1	18	1.6	0.221	0.59	0.454	0.23	0.635	0.93	0.345
Wind (W)	2	273	1.09	0.335	2.14	0.119	0.24	0.785	1.74	0.163
L x W	2	273	2.08	0.151	0.31	0.738	1.84	0.325	0.09	0.991
c) Model ‘Species’										
Aboveground traits	numDf	denDf	Height		LA		SLA		LDMC	
			*F*	*P*	*F*	*P*	*F*	*P*	*F*	*P*
Species (S)	24	299	108.2	**< 0.001**	92.85	**< 0.001**	111.6	**< 0.001**	34.04	**< 0.001**
Wind (W)	2	299	19.33	**< 0.001**	1.57	0.211	2.06	0.108	0.28	0.759
S x W	48	299	1.94	*0.081*	1.27	0.125	0.71	0.920	1.15	0.251
Root traits	numDf	denDf	SRL		SRSA		AD		RTD	
			*F*	*P*	*F*	*P*	*F*	*P*	*F*	*P*
Species (S)	24	151	14.55	**< 0.001**	7.00	**< 0.001**	33.39	**< 0.001**	10.40	**< 0.001**
Wind (W)	2	151	43.48	**< 0.001**	6.20	**0.003**	6.13	**0.003**	19.58	**< 0.001**
S x W	48	151	1.07	0.364	0.87	0.702	0.85	0.743	0.96	0.560
Biomass / allocation	numDf	denDf	Shoot		Root		Total		R:S	
			*F*	*P*	*F*	*P*	*F*	*P*	*F*	*P*
Species (S)	24	299	135.5	**< 0.001**	75.13	**< 0.001**	43.58	**< 0.001**	18.75	**< 0.001**
Wind (W)	2	299	0.99	0.375	0.61	0.542	0.09	0.916	1.01	0.367
S x W	48	299	0.94	0.579	1.31	0.102	1.12	0.108	0.73	0.897

**Table 2:** Information on plant species belonging to different growth forms (i.e., trees, grasses and forbs) used in the experiment and average plant height (in cm) of plant species measured at harvest at the different wind treatment levels [i.e., ‘Control’ (i.e., no wind), ‘Medium wind’ intensity and ‘High wind’ intensity] (a) as well as abiotic conditions in the greenhouse at the different wind treatment levels (b). In Table (a) plant age refers to the number of days since germination at the end of the experiment. In Table (a) and (b) *P* values represent results of ANOVAs that tested for differences between wind treatment levels for every species. In Table (a) different letters indicate significant differences between wind treatment levels after a Tukey HSD test. In Table (a) and (b) data represent mean ± SE (*n* = 5)

a)								
Growth form	Life history	Family	Species	Plant age (d)	Plant height (cm)			
					Control	Medium Wind	High Wind	*P*
Tree	Perennial	Fagaceae	*Fagus sylvatica* L.	140–143	11.5 ± 0.8	10.8 ± 0.8	13.5 ± 0.5	n.s
Tree	Perennial	Pinaceae	*Pinus sylvestris* L.	168–171	13.1 ± 0.7	14.2 ± 0.6	13.3 ± 0.7	n.s
Tree	Perennial	Platanaceae	*Platanus x hispanica* Münchh.	91	16.6 ± 0.6	17.2 ± 0.8	14.9 ± 0.4	n.s
Tree	Perennial	Sapindaceae	*Acer pseudoplatanus* L.	141–145	14.3 ± 0.7 a	12.9 ± 0.8 ab	10.3 ± 1.2 b	0.038
Tree	Perennial	Taxaceae	*Taxus baccata* L.	170–173	7.9 ± 0.4	7.2 ± 0.8	8.8 ± 0.7	n.s
Grass	Perennial	Juncaceae	*Luzula campestris* (L.) DC.	92	8.2 ± 2.0	7.3 ± 0.8	7.0 ± 0.6	n.s
Grass	Perennial	Poaceae	*Agrostis capillaris* L.	91	34.4 ± 1.7	37.3 ± 5.0	29.7 ± 0.8	n.s
Grass	Perennial	Poaceae	*Cynosurus cristatus* L.	91	30.1 ± 2.3	27.8 ± 5.2	22.8 ± 1.5	n.s
Grass	Perennial	Poaceae	*Dactylis glomerata* L.	92	43.6 ± 3.6	43.8 ± 2.7	39.7 ± 4.0	n.s
Grass	Perennial	Poaceae	*Festuca brevipila* R.Tracey	91	30.1 ± 0.6	26.3 ± 2.4	23.5 ± 1.7	n.s
Grass	Perennial	Poaceae	*Holcus lanatus* L.	91	31.2 ± 1.7	32.3 ± 2.3	29.4 ± 2.1	n.s
Grass	Perennial	Poaceae	*Poa pratensis* L.	91	38.0 ± 4.7	33.8 ± 5.1	31.1 ± 2.4	n.s
Grass	Annual	Poaceae	*Secale cereale* L.	91	37.1 ± 2.7	35.3 ± 1.8	31.0 ± 1.6	n.s
Grass	Annual	Poaceae	*Triticum aestivum* L.	91	33.2 ± 0.9	30.8 ± 2.2	30.3 ± 2.9	n.s
Grass	Annual	Poaceae	*Zea mays* L	91	75.0 ± 2.1 a	65.4 ± 1.1 b	62.2 ± 2.4 b	0.001
Forb	Annual	Asteraceae	*Conyza canadensis* (L.) Cronquist	91	25.4 ± 1.6	25.9 ± 2.5	22.7 ± 1.6	n.s
Forb	Perennial	Asteraceae	*Achillea milleflolium* L.	92	20.0 ± 1.8	19.8 ± 2.9	17.0 ± 0.6	n.s
Forb	Annual	Brassicaceae	*Lepidium sativum* L.	91	34.5 ± 3.1	32.9 ± 2.7	30.8 ± 3.3	n.s
Forb	(Bi-)Annual	Brassicaceae	*Brassica napus* L.	91	19.9 ± 1.1 a	14.5 ± 1.1 b	17.3 ± 0.9 ab	0.012
Forb	Annual	Fabaceae	*Trifolium dubium* Sibth.	92	21.4 ± 3.9 a	12.4 ± 1.6 ab	10.8 ± 1.4 b	0.027
Forb	Perennial	Fabaceae	*Trifolium pratense* L.	92	22.3 ± 1.1	19.4 ± 2.2	16.9 ± 1.1	n.s
Forb	Annual	Linaceae	*Linum usitatissimum* L.	91	35.2 ± 1.4	32.8 ± 3.2	34.5 ± 0.5	n.s
Forb	Perennial	Plantaginaceae	*Veronica chamaedrys* L.	92	21.4 ± 2.3	16.8 ± 3.4	13.8 ± 1.5	n.s
Forb	Perennial	Polygonaceae	*Rumex acetosella* L.	91	19.4 ± 7.8	12.4 ± 1.0	12.5 ± 0.4	n.s
Forb	Perennial	Rosaceae	*Sanguisorba minor* Scop.	92	10.5 ± 0.7	11.4 ± 0.8	9.8 ± 0.6	n.s
b)								
Abiotic conditions in the greenhouse				Control	Medium Wind		High Wind	*P*
Air temperature (°C)				24.9 ± 0.04	24.9 ± 0.05		24.9 ± 0.05	n.s
Air humidity (%)				55.3 ± 0.13	55.3 ± 0.14		55.2 ± 0.14	n.s

**Fig. 1:**
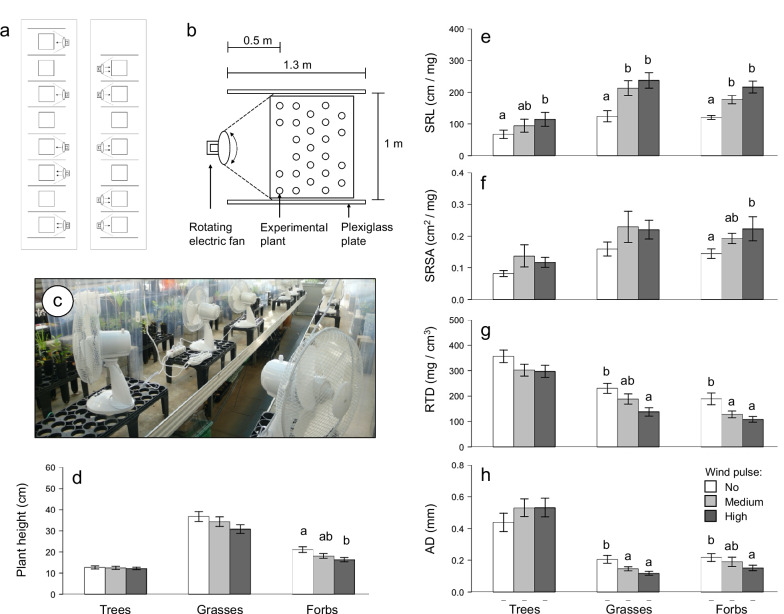
Schematic description of the experimental set-up (**a**, **b**), picture of the experiment (**c**) and results on measured plant height (**d**) and morphological root traits (**e**–**h**). **a** Spatial arrangement of the wind treatments in the greenhouse experiment. Overall, there were 5 replicate blocks for each wind treatment [‘no wind’ (0 m s^−1^), ‘medium wind intensity’ (0.9 m s^−1^) and ‘high wind intensity’ (1.8 m s^−1^)]. Blocks including experimental plants and rotating electric fans with the respective wind intensity level were shifted weekly during the experiment to reduce potential differences in microclimate within the greenhouse and edge effects. Arrows indicate wind intensity level (one arrow = medium wind intensity; two arrows = high wind intensity). **b** Arrangement of the electric fan and the experimental plants within one block. Blocks were separated using plexiglass plates (1.3 m × 1.5 m). Electric fans run for 30 min two times per day, turning from side to side to apply wind for all plants. To prevent edge effects and potential differences in wind intensity within blocks, pots with experimental plants were also shifted within blocks weekly (see Materials and methods section for further description). **c** Picture from the experiment showing the arrangement of electric fand and experimental plants in the greenhouse. **d** Average plant height, **e** specific root length (SRL), **f** specific root surface area (SRSA), **g** root tissue density (RTD) and **h** average root diameter (AD) of the three growth forms (trees, grasses and forbs) when grown under no (white), medium (light grey) and high (dark grey) wind pulse intensities. Data in (**d**–**h**) represents mean ± SE [in (**d**): *n* = 25 for tree species and *n* = 50 for grasses and forbs, whereas in (**e**), (**f**), (g) and (**h**): *n* = 15 for tree species and *n* = 30 for grasses and forbs]. Letters above bars indicate significant differences in morphological shoot and root traits between the three wind treatments within functional groups after a Tukey honest significant difference test

In contrast to shoot morphological traits, wind stress by short wind pulses of different intensity influenced root morphological traits across all growth forms (specific root length (SRL): *F*_2,191_ = 40.91, *P* < 0.001; specific root surface area (SRSA): *F*_2,191_ = 6.00, *P* = 0.003; average diameter (AD): *F*_2,191_ = 5.66, *P* = 0.004; root tissue density (RTD): *F*_2,191_ = 18.43, P < 0.001; Table 1a). For trees, grasses and forbs, SRL and SRSA increased, whereas RTD decreased when exposed to increasing wind pulse intensities (Fig. 1e, f, g). Plants of different growth forms showed similar responses in root morphological traits in response to stimulation by wind (‘growth form x wind’ interaction for SRL, SRSA and RTD: *P* > 0.3; Table 1a, Fig. 1e, f, g), except for AD, which slightly increased with increasing wind pulse intensity for trees, but significantly decreased in grasses and forbs (*F*_4,191_ = 4.19, *P* = 0.003; Table 1a; Fig. 1h). Similarly, root morphological traits of annuals vs. perennials and in general across species were also impacted by wind stress, whereas responses were similar across life-history types and species (‘life history x wind’ and ‘species x wind’ interactions for all root morphological traits: *P* > 0.1; Table 1b, c). These results suggest that wind stress by short wind pulses of different intensity promoted the development of thinner roots with increased SRL and SRSA. Besides effects on water and nutrient acquisition (Bardgett et al. 2014), such changes in root morphology are suggested to increase anchorage (Burylo et al. 2009; Werger et al. 2020), as higher root length is assumed to provide better mechanical support (see Zhang et al. 2021). Furthermore, the decreased RTD under wind action suggests that plants invest less root biomass to obtain given levels of surface area and length and, thus, have greater root–soil contact at less cost (Werger et al. 2020). Although in older trees and forbs tap roots contribute most to anchorage strength (Yang et al. 2016), it is suggested that anchorage strength of plants also depend on root-soil contact surface (i.e., root system size; Schutten et al. 2005), which is influenced by SRSA of a root. However, although our results might indicate that wind stress impacts root morphology in young plants and thus potentially promoting anchorage, further explicit mechanical tests are needed to verify wind effects on root anchorage.

While the wind-pulse related increase in SRL was significant for all growth forms, the increase in SRSA was only significant in forbs and marginal for trees and grasses (Fig. 1e, f). Trees showed only a marginal decrease in RTD in response to wind, whereas RTD for grasses and forbs significantly decreased with increasing wind pulse intensity (Fig. 1g). Although different in strength, we found similar responses in wind-induced root morphological traits across growth forms. This might be due to the fact that we tested wind effects in young plants (few-month old) whose microfibril structure and content as well as overall lignification, thus putative responsiveness to wind, might have been more similar compared to the structural differences of older plants (Teng et al. 2021). The overall weaker responses in tree species could possibly be explained by our experimental procedure, because we used few-monthly old tree saplings in four of five species. These saplings were exposed to outdoor conditions prior the experiment and might have been primed to wind stress, and may have contained more lignin to increase wind resistance (Cipollini 1997).

Beside morphological traits, we also tested whether short wind pulses of different intensity affected growth conditions and found no effect on air temperature and humidity (Table 2b), nor on biomass production and allocation patterns (i.e., root:shoot ratio; see Table 1). This suggests that, although not directly measured in this experiment, CO_2_ uptake and assimilation rate, which also depends on abiotic conditions like temperature, were not affected by short wind pulses. Furthermore, we did not observe a change in root:shoot ratios with increasing wind pulse intensities, which may suggest that such effects are only found when plants were exposed to longer or more continuous wind actions (e.g., Gardiner et al. 2016; Feng et al. 2019) or that the speed of wind pulses in this experiment might have been too slow to cause responses in biomass production and allocation.

However, wind stress changed root morphology in this experiment. As plant responses in roots are suggested to depend on the plant height (James et al. 2006) we afterwards tested whether the height of a plant species affected responses in root traits to short wind pulses. This analysis revealed that the plant height per species in this experiment was weakly positively correlated with changes in SRL and weakly negatively correlated with changes in AD (Fig. 2), whereas SRSA and RTD were not correlated with plant height. When exposed to both medium and high wind pulse intensities, species with taller shoots in this experiment (i.e., grasses and forbs) experienced a larger increase in SRL and a larger decrease in AD in comparison to control plants than species with shorter shoots (i.e., mainly tree species; Fig. 2). These results suggest that species with taller shoots, which are likely to experience stronger wind-induced oscillations (Sellier and Suzuki 2020), might have experienced stronger thigmomorphogenesis effects in roots, possibly due to stretch-activated responses in cell membranes (Telewski 2021) and associated changes in phytohormone concentrations such as auxin, ethylene, cytokinins and abscisic acid, which are known to regulate root elongation and branching (Lee et al. 2018; Telewski 2021).

**Fig. 2:**
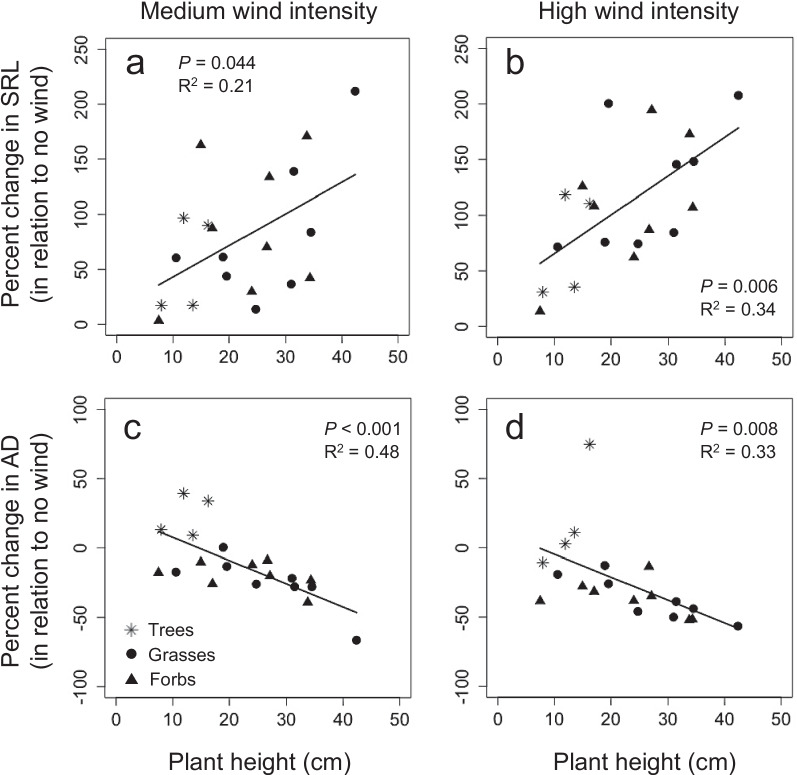
Relationship between average plant height of trees (asterisks), grasses (circles) and forbs (triangles) in this experiment and percent change in specific root length (SRL) and average diameter (AD) between control plants (i.e., no wind) and medium wind intensity (SRL: **a**; AD: **c**) as well as high wind intensity (SRL: **b**; AD: **d**). *P*- and *R*^2^ values indicate results of linear regressions

Taken together, our study for the first time reveals a clear pattern of wind response in young plants: across growth forms, wind stress by short wind pulses has little effect on shoot morphology, but consistently changes root morphology to possibly strengthen anchorage in a plant height-dependent manner. Further tests on these effects would be helpful to understand plant responses during development and in interaction with environmental stresses, such as climate change, where wind effects are expected to increase.

## Materials and methods

### Study species

To test whether species of different growth form differ in morphological traits in response to wind pulses we selected 25 plant species belonging to three main groups: trees, grasses and forbs (Table 2a). Seeds of the species were either collected by hand on the area of the Botanical Garden of the University of Potsdam (N52°24′28.31ʺ, E13°1′15.71ʺ; Brandenburg; Germany) and agricultural fields (N53°14′17.16ʺ, E12°12′8.56ʺ; Brandenburg; Germany), or purchased (*Lepidium sativum*; Carl Pabst Samen & Saaten GmbH; Germany). Except seeds of *Platanus* x *hispanica*, seeds of the woody species failed to germinate prior the experiment. Therefore, we collected few-month old, similar-sized saplings of the four remaining tree species on the area of the Botanical Garden of the University of Potsdam.

### Experimental preparation

Seeds were germinated in a staggered design on washed sand (grain size: 2 mm; Brun & Böhm; Potsdam, Germany) in sterile plastic chambers (32 × 50 × 14 cm; Meyer, Rellingen, Germany) in a greenhouse at the University of Potsdam (min/max: temperature 15 °C/25 °C; relative humidity 33%/90%; additional light: 140 μmol * s^−1^ * m^−2^; 12/12 h light/dark) in May 2020, to obtain coeval seedlings. Tree saplings were collected in late April 2020 and grown in sterile plastic chambers in a soil:sand mixture prior to usage in the experiment. The soil:sand mixture consisted of a 1:1 mixture of sieved (mesh size: 5 mm) native soil collected from the field site of the University of Potsdam and washed sand. This soil:sand mixture (12.02 mg kg^−1^ NH_4_^+^, 20.81 mg kg^−1^ NO_3_^−^, 1.15 mg kg^−1^ P-Olsen) was also used in the experiment as potting medium to facilitate the washing of roots afterwards.

Pots (Deepots D25L: volume 0.41 L; height 25 cm; diameter 5 cm; Stuewe & Sons, Tangent, Oregon, USA) were filled with the soil:sand mixture to ensure that experimental plants received equal growth conditions (i.e., standardized soil volume for root growth and similar soil nutrients). Pots were filled to same height (0.5 cm below pot edge) to enable similar wind effects between experimental plants.

In June 2020, 2-week-old, similar-sized seedlings and similar-sized tree saplings were planted in the prepared pots, with one individual seedling/sapling per pot. After planting, before the wind treatment (see below) young seedlings and saplings were allowed to acclimatize for 2 weeks.

### Wind treatment

We performed a wind intensity treatment using wind tunnels. These wind tunnels (100 × 130 × 150 cm) were created on greenhouse desks and separated using transparent Plexiglass plates (Salux GmbH, Saxoni-Anhalt, Germany). To expose experimental plants to different wind pulse intensities wind tunnels were equipped with electric fans (OK desk fan OTF3331W, Imtron GmbH, Bavaria, Germany) or no fans for control plants without wind stimulus. Electric fans were placed at the height of the pots, spaced 50 cm apart from the experimental plants. Wind speed by electric fans was set to medium and high levels to obtain, together with the no fan treatment, three different wind pulse levels. Wind speeds were measured using an anemometer (Profi-Wind gauge, Bresser GmbH, Germany) at 20 cm height. Taken together, the wind treatment consisted of three wind pulse intensity levels: 1) no wind (0 m s^−1^), 2) medium wind intensity (0.9 m s^−1^) and 3) high wind intensity (1.8 m s^−1^).

Electric fans were run for half an hour, twice per day (9 to 9:30 am and 7 to 7:30 pm), to investigate recurring short-term wind effects (de Langre 2008) and to avoid differences in abiotic conditions such as temperature, humidity, and CO_2_ concentration between wind treatment levels that might affect biomass production (Retuerto and Woodward 1992).

### Experimental set up

In the End of June 2020, planted pots were distributed to the wind treatments and were arranged in a randomized block design, where each block contained one wind pulse intensity level and one single replicate per species. Each species x wind level was replicated 5 times resulting in 375 pots (25 species × 3 wind pulse intensities × 5 replicates). In blocks, pots were placed in trays and spaced at least 10 cm apart in a staggered arrangement to enable wind flow for all experimental plants. To reduce potential differences in microclimate within the greenhouse blocks were shifted weekly. In addition, to reduce edge effects and potential differences in wind intensity within blocks, pots were also shifted within blocks weekly. Furthermore, soil water content was kept constant (controlled by weighing) by watering the pots with tap water every third day to avoid confounding effects of wind intensity on soil water content via possible differences in evapotranspiration. The experiment was run for 9 weeks to observe wind effects in young plants and to prevent roots becoming pot-bound (personal observations, from pre-experiments; J. Heinze).

### Measurements

During the experiment, air temperature and relative air humidity were measured in all three wind treatments using HOBO Pro v2 data loggers (Onset Computer, Bourne, Massachusetts, USA) to test for potential differences in abiotic conditions between the different wind intensity levels.

After 9 weeks (shortly before harvest), shoot traits were measured in accordance to Pérez-Harguindeguy et al. (2013). Immediately before taking trait measurements, pots were watered to ensure that plants were fully turgescent. For every experimental plant, we determined plant height, counted all leaves, and randomly sampled one of the youngest fully expanded and healthy leaves. Immediately after sampling, leaves were weighed and scanned (Epson Expression 1680, Seiko Epson Corporation, Suwa Nagano, Japan), and LA was measured using ImageJ. Leaves were then dried (48 h, 80 °C) and weighed. Afterwards, LDMC (mg g^−1^) and SLA (cm^2^ g^−1^) were calculated.

Following shoot trait measurements, aboveground biomass was harvested and afterwards, roots were carefully washed. To investigate whether wind impacted root morphological traits of plants (i.e., growth forms), a subset of individuals of each species (three replicates per wind pulse intensity level; i.e., 225 samples in total) was analyzed. A representative subsample (maximum diameter: 0.9 mm) of the whole root system of each plant was analyzed using the WinRhizo scanner-based system (Regents Instruments, Inc., Canada) to determine root diameter and length. Afterwards, roots were dried (48 h, 80 °C) and weighed to obtain root mass of the subsample. Specific root morphological traits (except AD) were calculated: SRL (cm mg^−1^), SRSA (cm^2^ mg^−1^) and RTD (mg cm^−3^). For the calculation of RTD, we summed the volume of 0.1 mm diameter classes as recommended by Rose (2017). Shoot and root biomass of all experimental plants was dried (48 h, 80 °C) and weighed to assess root:shoot ratio.

### Statistical analysis

All analyses were performed in R version 3.6.3. Prior to analysis, residuals were checked for homogeneity of variance and tested for normality.

To test whether wind and its intensity affected biomass production and allocation, as well as shoot and root morphological traits for plants of different growth forms (i.e., trees, grasses and forbs) we performed ANOVAs. To account for the nesting of species within growth forms and the unbalanced sample size between growth forms (tree species: *n* = 5; grass and forb species: each *n* = 10), we analyzed the data with linear mixed effects models using the “lme4” package. We used the lmerTest function to estimate *P*-values and degrees of freedom with type III Kenward-Roger approximation.

The model included the predictors ‘growth form’ (tree, grass, forb), ‘wind pulse level’ (‘no wind’ vs. ‘medium’ vs. ‘high’ wind intensity) as well as their interaction. The model tested the effects of the predictors and their interactions on morphological traits of shoots (i.e., plant height, LDMC, SLA and LA), and roots (SRL, SRSA, AD and RTD) as well as on biomass production (shoot, root, total) and root:shoot ratio. In the model, ‘species’ was included as random effect. To test for differences between wind pulse intensities in morphological traits within growth forms we performed Tukey HSD tests using the multcomp package. Similar models and analyses tested for wind effects between annuals vs. perennials within herbaceous plants (i.e., ‘Life history’) and for all ‘Species’.

Furthermore, we tested whether plant height affected changes in root morphological traits. Therefore, for each species we calculated‚ the relative change (in percent) in root morphological traits between control plants (i.e., no wind) and medium respectively high wind pulse intensity.

$$\mathrm{Change\, in\, morphological\, trait\, X}=(({\mathrm{X}}_{\mathrm{wind\, pulse \,level}}*100)/{\mathrm{X}}_{\mathrm{control}} )-100;$$

where X _wind pulse level_ is the mean value of a given root morphological trait of a species at either medium or high wind pulse intensity and X _control_ is the same root morphological trait of this species at control conditions (i.e., no wind).

This measurement was then related to average plant height per species realized in this experiment. As wind impacted plant height in four species, we excluded these species (*Acer pseudoplanatus* L., *Brasscia napus* L., *Trifolium dubium* Sibth. and *Zea mays* L*.*; see Table 2a) from this analysis to avoid confounding by wind. The relationships between changes in root morphological traits and plant height were analyzed using linear regressions.

## Data Availability

Data are available at: https://doi.org/10.6084/m9.figshare.24145416.v1.

## Abbreviations

Height: Plant height (cm)
LA: Leaf area (cm^2^)
SLA: Specific leaf area (cm^2^ g^–1^)
LDMC: Leaf dry matter content (mg g^–1^)
SRL: Specific root length (cm mg^–1^)
SRSA: Specific root surface area (cm^2^ mg^–1^)
RTD: Root tissue density (mg cm^–3^)
AD: Average diameter (mm)

## References

[CR1] Anten NPR, Alcalá-Herrera R, Schieving F, Onoda Y (2010). Wind and mechanical stimuli differentially affect leaf traits in Plantago major. New Phytol.

[CR2] Bardgett RD, Mommer L, de Vries FT (2014). Going underground: root traits as drivers of ecosystem processes. Trends Ecol Evol.

[CR3] Burylo M, Rey F, Roumet C, Buisson E, Dutoit T (2009). Linking plant morphological traits to uprooting resistance in eroded marly lands (Southern Alps, France). Plant Soil.

[CR4] Cipollini DF (1997). Wind-induced mechanical stimulation increases pest resistance in common bean. Oecologia.

[CR5] De Langre E (2008) Effects of wind on plants. Annu Rev Fluid Mech 40:141–168. 10.1146/annurev.fluid.40.111406.102135

[CR6] Feng J, Huang P, Wan X (2019). Interactive effects of wind and light on growth and architecture of poplar saplings. Ecol Res.

[CR7] Gardiner B, Berry P, Moulia B (2016). Review: Wind impacts on plant growth, mechanics and damage. Plant Sci.

[CR8] James KR, Haritos N, Ades PK (2006). Mechanical stability of trees under dynamic loads. Am J Bot.

[CR9] Larson JA, Funk JL (2016). Seedling root responses to soil moisture and the identification of belowground trait spectrum across three growth forms. New Phytol.

[CR10] Lee S, Sergeeva LI, Vreughenhil D (2018). Natural variation of hormone levels in *Arabidopsis* roots and correlations with complex root architecture. Int J Plant Biol.

[CR11] Liu C, Li Y, Chen L, He N (2019). Variation in leaf morphological, stomatal, and anatomical traits and their relationships in temperate and subtropical forests. Sci Rep.

[CR12] Pérez-Harguindeguy N, Diaz S, Garnier E (2013). New handbook for standardized measurement of plant functional traits worldwide. Austral J Bot.

[CR13] Retuerto R, Woodward FI (1992). Effects of windspeed on the growth and biomass allocation of white mustard *Sinapis alba* L. Oecologia.

[CR14] Rose L (2017). Pitfalls in root trait calculations: how ignoring diameter heterogeneity can lead to overestimation of functional traits. Front Plant Sci.

[CR15] Schutten J, Dainty J, Davy J (2005). Root anchorage and its significance for submerged plants in shallow lakes. J Ecol.

[CR16] Sellier D, Suzuki S (2020). Age dynamics of wind risk and tree sway characteristics in a softwood plantation. Front For Glob Change.

[CR17] Telewski FW (2021). Mechanosensing and plant growth regulators elicited during the thigmomorphogenetic response. Front For Glob Change.

[CR18] Teng RM, Wang Y-X, Li H, Lin S-J, Liu H, Zhuang J (2021). Effects of shading on lignin biosynthesis in the leaf of tea plant (*Camellia sinensis* (L.) O. Kuntze). Mol Genet Genomics.

[CR19] Werger L, Bergmann J, Weber E, Heinze J (2020). Wind intensity affects fine root morphological traits with consequences for plant-soil feedback effects. AoB Plants.

[CR20] Yang M, Défossez P, Danjon F, Dupont S, Fourcaud T (2016). Which root architectural elements contribute the best to anchorage of *Pinus* species? Insigths from in silico experiments. Plant Soil.

[CR21] Zhang S, Liu G, Cui Q, Huang Z, Ye X, Cornelissen JHC (2021). New field wind manipulation methodology reveals adaptive responses of steppe plants to increased and reduced wind speed. Plant Methods.

